# General and comparative self-rated health in chronic stroke: an important outcome measure for health professionals

**DOI:** 10.1186/s12883-022-02592-7

**Published:** 2022-03-07

**Authors:** Ramon Távora Viana, Érika de Freitas Araújo, Lidiane Andrea Oliveira Lima, Luci Fuscaldi Teixeira-Salmela, Christina Danielli Coelho de Morais Faria

**Affiliations:** 1 Department of Physical Therapy, Faculty of Medicine, Universidade Federal Do Ceará, Fortaleza, Brazil; 2 Department of Physical Therapy, Universidade Federal de Minas Gerais, Av. Antônio Carlos, 6627, Campus Pampulha, 1270-901, MG Belo Horizonte, Brazil

**Keywords:** Self-rated health, Health assessment, Stroke, Affective symptoms, Motor disorders, Physical activity

## Abstract

**Background:**

After a stroke, several aspects of health and function may influence how individuals perceive their own health. However, self-rated health (SRH), as well as its relationship with functioning, has been little explored in individuals with stroke. The aims of this study were to determine how individuals with chronic post-stroke disabilities evaluate their health, considering general, time- and age-comparative SRH questions and to investigate whether SRH measures would be influenced by the following health and functioning domains: mental/physical functions and personal factors.

**Methods:**

Sixty-nine individuals with chronic post-stroke disabilities answered the three types of SRH questions and were assessed regarding depressive symptoms (emotional function domain), physical activity levels (physical function domain), and engagement in physical activity practice (personal factor domain). Subjects were divided into the following groups: good/poor for the general SRH question; better, similar, and "worse" for both time- and age-comparative questions. Between-group differences in the three domains for each SRH question were investigated (α = 5%).

**Results:**

General SRH was rated as good by 73% of the participants. Time- and age-comparative SRH was rated as better by 36% and 47% and as similar by 31% and 28% of the subjects, respectively. Significant between-group differences in emotional function were found for both the general and age-comparative questions. For the time-comparative question, significant differences were only observed for physical function.

**Conclusion:**

SRH evaluation differed in individuals with chronic post-stroke disabilities according to the types of questions and health/functioning domains.

## Background

According to the World Health Organization (WHO), health is defined “as a state of complete physical, mental, and social well-being” [1]. Functioning is an important component of health and has an essential role in health status [2, 3]. In addition, rehabilitation professionals are important members of the healthcare team and the primary goal of rehabilitation, as a health-related intervention, is functioning [4].

As stated by Frier et al., “following the acquisition of disability, there is a decline in social determinants of health”, which increases morbidity and mortality [3]. Since stroke has a high prevalence of disability, several aspects of health and function may influence how individuals perceive their own health. However, which and how these aspects could influence individuals’ health are not clear [5]. Previous studies reported that up to 90% of the stroke survivors live with some degree of disability [6], such as emotional impairments [7], limited-community ambulation, and social participation restrictions [7]. These findings emphasize the need for continuous health monitoring of individuals with stroke by the rehabilitation team.

Self-rated health (SRH) questions are simple, valid, and recommended indicators for continuous monitoring health status [8] and are widely used, due to their power in predicting comorbidities and mortality [9, 10]. The use of SRH reduces observation and measurement bias, facilitates data analyses, and reduces the costs of data collection [11, 12]. Finally, SRH provides valuable information on health, function, and risk factors for developing stroke [13]. SRH is a patient-reported outcome, often evaluated using a single question, in which individuals choose between four or five response items [14]. The question can be general (“In general, would you say your health is: excellent, very good, good, poor, and very poor?”) [15], time-comparative (“Compared to one year ago, would you say your health is: much better, somewhat better, about the same, somewhat worse, or much worse?”) or age-comparative (“Compared to other people of your age, you would say your health is better, similar, or worse?”) [16]. These questions refers to different elements of health and have specific applications [17].

In general, SRH determinants are reported as elements of functioning, such as the mental and physical domains, and personal factors [18]. These relationships have been previously observed with the elderly, as the domains of functioning were found to be determinants of SRH [19]. Several aspects of health and functioning may influence how individuals perceive their own health [2, 5, 18, 20–27]. However, there is little knowledge of how people with chronic post-stroke disabilities rate their health, considering different types of SRH questions, and whether there would be differences in emotional and physical functions, as well as in personal factors, when they rate their health based upon general, time- and age-comparative questions. Therefore, the aims of the present study were to determine how individuals with chronic stroke evaluate their health, considering general, time-and age-comparative questions and to investigate whether SRH measures would be influenced by the following health and functioning domains: mental/physical functions and personal factors.

## Methods

A cross-sectional, exploratory study was conducted with individuals with stroke from six basic health-care units of the Public Health Care System of two Brazilian metropolitan cities. This study was approved by the institutional research ethical committees of both the Universidade Federal de Minas Gerais and the Municipal Health Secretariat of the cities of Belo Horizonte and Fortaleza (#CAAE: 14,038,313.4.0000.5149). All methods were performed in accordance with the relevant guidelines and regulations. Informed consent to participate in the study was obtained from all participants (or from their legal guardian in the case of illiterate participants) prior to data collection, by signing the consent form approved by the ethical committees. Data were obtained from the patients’ medical records at the basic health-care unities and the participants were evaluated at their homes.

In Brazil, individuals with chronic disabilities are commonly assisted by health professionals, who work in decentralized basic health-care units. These units are located in low-income communities and typically serve individuals, who depend only on the public health system [28].

### Participants

All individuals, who have had a stroke and were identified by the health professionals of the six basic health-care unities were invited to participate, based upon the following criteria: had a clinical diagnosis of a primary or recurrent stroke for more than six months; were living in the area and were users of the public health-care unities, due to chronic disabilities; and were ≥ 20 years of age. Individuals were excluded if they had motor or sensory aphasia and cognitive impairments, which were determined by the education-adjusted cut-off scores on the Mini-Mental State Examination [29].

### Outcomes

#### Self-rated health

SRH was measured using general, time-, and age-comparative questions, following recommendations of a recent systematic review on SRH after stroke [5]. SRH questions have good reproducibility, moderate reliability, and strong concurrent and discriminant validities, when compared to the Short-Form health survey-12 (SF-12) [30].

General SRH was measured using the following first question of the Short-form health survey (SF-36): “In general, would you say your health is: excellent, very good, good, poor, and very poor?” [31]. Participants were divided into two groups: good SRH, by collapsing the excellent, very good, and good responses and poor SRH, by collapsing the poor and very poor responses, as applied in previous studies [32, 33]. Such transformation is simple, improves statistical analyses, allows for keeping respondents’ answers in their original response options (instead of dichotomizing response options), and improves the interpretation of mean SRH values in populations groups [32].

Time-comparative SRH was evaluated using the second question of the SF-36 as follows: “Compared to one year ago, would you say your health is: much better, somewhat better, about the same, somewhat worse, or much worse?” [31]. Participants were divided into three groups: better SRH, by collapsing the much better and somewhat better responses, similar (“about the same” response), and worse, by collapsing the somewhat worse and much worse responses, as also used in previous studies with individuals with stroke [34].

Age-comparative SRH was assessed by asking the participants the following question: “Compared to other people of your age, you would say your health is better, similar, or worse?”. Based upon their responses, the participants were divided into three groups as used in previous studies with individuals with stroke [35].

#### Assessment of health domains

The following criteria were used to select the outcomes, to characterize the domains related to emotional and physical functions, as well as the personal factors: be commonly pointed-out as associated with SRH in various populations [14, 18, 36]; be an important outcome for stroke rehabilitation recommended by clinical practice guidelines [37]; and have the potential to be improved or modified with rehabilitation interventions [37].

Emotional function was characterized by the presence of depressive symptoms, which was assessed by the short version of the Geriatric Depression Scale. The Geriatric Depression Scale has been supported psychometrically and used with various populations, including individuals with stroke [38, 39]. The Geriatric Depression Scale contains 15 items, which are answered using a yes/no format and the scores range from 0 to 15, with higher scores reflecting more symptoms of depression [38]. A cut-off score of six points is used as the threshold, to discriminate individuals with and without depressive symptoms [40]. The total scores were registered for analyses.

Physical function was characterized by the level of physical activity, which was assessed by the Human Activity Profile adjusted activity scores [41]. The Human Activity Profile is a survey that includes 94 activities, such as self-care, transportation, home maintenance, entertainment/social, and physical exercises, which are sequentially rated according to their required metabolic equivalents. For each item, there are three possible responses: “still doing the activity”, “have stopped doing the activity”, and “never did the activity”. The administration and scoring procedures followed recommended protocols and scores are tallied to provide a maximum activity score, which indicated the highest metabolic equivalent activity the person still performs [42]. The adjusted activity scores, which was registered for analyses, was determined by subtracting the number of activities that the respondent had discontinued performing from the maximum score and indicated the average typical metabolic equivalent levels [41]. The Human Activity Profile has shown to be reliable and valid to be used with individuals with stroke [42].

Personal factors were characterized by the participant’s engagement in physical activity practice, based upon the classification of the Centers for Disease Control and Prevention [43]. According to the frequency, duration, and intensity of the estimated metabolic expenditure of exercise(s) performed over the last four weeks, the individuals may be classified as vigorous, moderate, insufficient, or inactive, as recommended by the Physical Activity Trends/United States. For analyses, the participants were classified as sedentary (inactive) and active (vigorous, moderate, or insufficient active) [43].

### Procedures

Potential subjects were identified by the health care team of the six basic health-care units. Their contact information and clinical and demographic data were extracted from their medical records and used for characterization purposes. Then, a home visit was scheduled and when the participants did not agree to participate, the collected information was discarded. During the home visits, the participants confirmed the collected data and added any missing information from the medical records. Then, they were asked to answer the general, time-, and age-comparative questions, followed by the administration of the Geriatric Depression Scale and the Human Activity Profile, as well as information on engagement in physical activity practice.

### Statistical analyses

Sample size calculation was estimated, based upon between-group comparisons (good and poor general SRH), using the G* power software (version 3.1.9.2) and considering a power of 95%, a significance level of 5%, and a β of 80%. For this calculation, preliminary data of 20 participants considering the main variables of interest, i.e., emotional function (the Geriatric Depression Scale scores), physical function (Human Activity Profile adjusted activity scores), and engagement in physical activity practice (classification of the Centers for Disease Control and Prevention), were considered. The sample size was based upon the highest result of the three investigated outcomes (the Geriatric Depression Scale scores, the Human Activity Profile adjusted activity scores, and engagement in physical activity practice). The sample size analysis of engagement in physical activity practice was the highest and resulted in 31 individuals per group. The following proportion values were used for this calculation: 21.1% (*n* = 4) active individuals with good and 0% (*n* = 0) with poor SRH; and 57.9% (*n* = 11) individuals classified as sedentary with good and 21.1% (*n* = 4) with poor SRH. Considering these results, a sample size of 62 participants was set.

Descriptive statistics, means, standard deviations (SD), medians, interquartile ranges (IQR), and frequencies were calculated. Tests for normality (Kolmogorov–Smirnov) were performed for all variables. To ensure that age, sex, and associated comorbidities, which are variables classically reported to affect SRH and stroke [19, 44, 45], did not influence the outcomes of interest, between-group differences were evaluated using chi-square and independent t-tests for the general SRH groups (good and poor). For both the time- and age-comparative questions (better, similar, and worse), these differences were evaluated using Kruskal Wallis test, one-way ANOVA, and Fisher's exact tests.

Three inferential analyzes were performed, considering the total Geriatric Depression Scale scores, the Human activity profile adjusted activity scores, and the classification on the engagement in physical activity practice. Between-group comparisons were carried-out, as follows: For the general question, Mann–Whitney-U tests were used. For the time- and age-comparative questions, Kruskal–Wallis tests were used, followed by post-hoc analyses using Mann–Whitney tests [46]. All analyzes were performed with the Statistical package for the social sciences for Windows (Version 17.0, SPSS Inc., Chicago, Illinois, USA) with a significance level of 5%.

## Results

### Participants

Out of the 174 potential individuals, 105 were excluded for the following reasons: cognitive impairments (*n* = 25), change of address (*n* = 29), no stroke diagnosis (*n* = 19), death (*n* = 13), aphasia (*n* = 7), refusals (*n* = 7), and acute post-stroke phases (*n* = 5). Thus, 69 were eligible and included. Their demographic and clinical information, including their levels of disability (Modified Rankin Scale) and motor impairments (Fugl-Meyer Assessment Scale) [47, 48], as well as the descriptive data on their general and comparative SRH questions are given in Table 1. The participants had a mean age of 66 (SD 12) years and a mean time since the onset of the stroke of 64 (SD 57) months. The groups were similar regarding age, sex, and associated comorbidities (0.10 ≤ *p* ≤ 0.89) (Table 1).

**Table 1:** Demographic and clinical characteristics of the participants

Characteristics		*n* = 69
Age (years), mean ± SD (min–max)		66 ± 12 (31–93)
Sex, n women (%)		38 (55.1)
Time since stroke (months), mean ± SD (min–max)		64.1 ± 56.9 (6–276)
Cognition (MMSE scores), median ± IQR (min–max)		21 ± 7 (13–30)
Associated comorbidities, n (%)	None	1 (1.5)
	One	7 (10.1)
	Two	6 (8.7)
	≥ 3	55 (79.7)
Education, n (%)	Illiterate	16 (23.2)
	Incomplete elementary school	19 (27.5)
	Complete elementary school	27 (39.1)
	High school	7 (10.2)
Levels of disability (Modified Rankin Scale), n (%)	No symptoms, no significant, or mild disability	18 (26.1)
	Moderate disability	38 (55.1)
	Moderately severe and severe disability	13 (18.8)
Motor impairments (Fugl-Meyer Assessment Scale: 0–100) n (%)	None	4 (5.8)
	Mild	7 (10.2)
	Moderately mild	25 (36.2)
	Severely moderate	20 (29)
	Severe (< 50)	13 (18.8)
General self-rated health, n (%)	Good	49 (73)
	Poor	18 (27)
Time-comparative self-rated health, n (%)	Better	24 (36)
	Similar	21 (31)
	Worse	22 (33)
Age-comparative self-rated health, n (%)	Better	32 (47)
	Similar	19 (28)
	Worse	17 (25)
GDS scores (0–15), median ± IQR (min–max)		6 ± 6.5 (0–14)
AAS (0–94), median ± IQR (min–max)		47 ± 36 (0–93)
Engagement in physical activity practice, n (%)	Active	18 (26.1)
	Sedentary	51 (73.9)

*SD* Standard deviation, *IQR* Interquartile range, *MMSE* Mini-Mental State Examination, *GDS* Geriatric Depression Scale, *AAS* Adjusted activity scores

### Between-group comparisons

Data regarding the comparisons between the emotional and physical functions, as well as the personal factor for the general and comparative SRH questions are reported in Table 2. For the general question, Mann–Whitney-U test revealed significant between-group differences only for emotional function (*p* < 0.001), showing that the participants who rated their health as good had lower Geriatric Depression Scale scores, ie, fewer depressive symptoms.

**Table 2:** Descriptive statistics and between-group comparisons regarding emotional/physical functions and personal factor for each type of self-rated health question

Self-rated health question	Groups	Emotional function (GDS),median ± IQR	Physical function (AAS) median ± IQR	Personal factor (engagement in physical activity practice)	
				Sedentary (%)	Active (%)
General	Good	5 ± 6^a^	51 ± 40^a^	50.7ª	22.4ª
	Poor	10 ± 5^b^	42 ± 25.75^a^	22.4ª	4.5ª
Age-comparative	Better	4 ± 6.5ª	47.5 ± 28.5ª	33.8ª	13.2ª
	Similar	6 ± 5^a^	53.5 ± 43ª	19.1ª	8.8ª
	Worse	10 ± 4^b^	41 ± 32ª	22.1ª	3.0ª
Time-comparative	Better	6.5 ± 5.5ª	59 ± 25ª	29.8ª	6ª
	Similar	5 ± 8ª	46 ± 36.8^b^	17.9ª	13.4ª
	Worse	8.5 ± 7.8ª	39 ± 25.5^b^	25.4ª	7.5ª

For each column, different letters (abc) represent significant between-group differences for each self-rated health question (p ≤ 0.05); *GDS* Geriatric depression scale, *AAS* Adjusted- activity score, *IQR* Interquartile range

For the age-comparative question, Kruskal–Wallis test also revealed significant between-group differences only found for emotional function (*p* < 0.001). Post-hoc analysis test revealed that these differences were significant only for the worse group, compared to the better and similar groups (*p* < 0.003). No significant differences were found between the better and similar groups (*p* = 0.09). These results showed that individuals, who rated their health as better and similar, had lower Geriatric Depression Scale scores, when compared with those who rated their health as worse. No significant between-group differences were found neither for physical function (0.11 ≤ *p* ≤ 0.58) nor for the personal factor (0.17 ≤ *p* ≤ 0.21).

For the time-comparative question, Kruskal–Wallis test showed significant between-group differences only for physical function (*p* = 0.03). Post-hoc analysis revealed that these differences were significant only for the better group, compared to the similar and worse groups (*p* < 0.04). No significant differences were found between the similar and worse groups (*p* = 0.89) These results showed that individuals, who rated their health as better had higher adjusted activity scores, compared with those who rated their health as similar and worse. No significant between-group differences were found neither for emotional function (*p* = 0.27) nor for the personal factor (*p* = 0.12).

## Discussion

The present study aimed at determining how individuals with chronic post-stroke disabilities evaluated their health, considering general, time-and age-comparative SRH questions and investigating whether SRH measures would be influenced by the following domains of health and functioning: mental/physical functions and personal factors. The results showed that despite having chronic disabilities, most of the participants rated their general health as good and their comparative health as better or similar. The SRH questions showed differences regarding emotional and physical functions. Individuals, who rated their general health as good, had fewer depressive symptoms than those who rated their general health as poor. For the age-comparative SRH question, those who rated their health as better or similar, had fewer depressive symptoms, when compared to those who rated their health as worse. For the time-comparative SRH question, individuals who rated their health as better, had higher levels of physical activity, when compared to those who rated their health as similar or worse. There were not found any significant between-group differences for any of the SRH questions, when engagement in physical activity practice was analyzed. Therefore, emotional and physical functions, which are commonly targeted during rehabilitation interventions, appear to influence SRH of individuals with chronic stroke.

Most individuals rated their general health as good and as better or similar for both time- and age-comparative questions. General SRH showed the most positive responses (73%), while the time-comparative SRH showed the most negative response (33%). Similar to the present findings, Skaner et al. [23] also reported that the majority of individuals reported their health as good or very good 12 months after the stroke, even in the presence of depressive symptoms.

Despite having chronic disabilities, individuals tend to have an optimistic view of their health. They seek reinforcement in aspects that agree with their positive views and ignore the factors that may alter the concept of health [18]. This finding was reported by Mavaddat as the ‘disability paradox’ in SRH [49]. Two distinct psychological processes may explain this. The first refers to the strategies for coping with the health condition, which remain unchanged until two years after the stroke [50]. Individuals possibly remain in denial and do not recognize stroke as an event that compromise their health [9]. The second refers to the cognitive process model for rating SRH proposed by Jylhä [14], in which other aspects of the individuals’ lives, which are not related to physical and mental impairments, such as contextual factors, tend to influence them to rate their health as good [51]. As previously reported, people who have long-term disabilities move towards disability acceptance and support [3], which may influence their own perceptions of health towards a positive view.

For both the general and age-comparative SRH questions, significant between-group differences were observed only for emotional function. On the other hand, for the time-comparative question, significant differences were observed only for physical function. Significant associations between general and age-comparative SRH and variables related to emotional function, such as depressive symptoms and life satisfaction were already reported with the elderly [17]. Associations between SRH and variables related to emotional function were also reported after stroke [52]. Emotional function, assessed by the Geriatric Depression Scale, was able to predict declines in activities of daily living after stroke [53] and represents the psychological aspect of post-stroke recovery [54]. Depressive symptoms are often observed after a stroke [7] and previous studies reported significant relationships between depressive symptoms and poor prognosis, poor adherence to treatment, and worse quality of life [55].

In the present study, individuals, who rated their general health as good and their age-comparative health as better or similar had fewer depressive symptoms. It is possible that rehabilitation interventions aiming at improving emotional function could have the potential to improve general and age-comparative SRH of individuals with stroke, but this should be further investigated.

For the time-comparative question, individuals who rated their health as better than before, had higher levels of physical activity, when compared to those who rated their health as similar or worse. However, this was not found for the age-comparative question. Although correlated, age- and time-comparative SRH should not be compared, since they were found to be weakly correlated and behave differently for specific health conditions with the elderly [56]. In the present study, the questions also showed different results regarding the emotional and physical functions. In addition, the present findings suggest that time- and age-comparative SRH should not be compared and used interchangeable for individuals with stroke, as previously recommended for the elderly [56].

Physical function, ie, level of physical activity, was the only variable that showed differences between the groups for the time-comparative SRH question. Individuals tend to increase their levels of physical activity over time and this occurs mainly during the first year after the stroke [57]. It is possible that even at the chronic stages, individuals maintain the expectation of increasing their levels of physical activity in relation to the previous year.

Surprisingly regarding the personal factor, which was characterized by engagement in physical activity practice, there were not found any significant between-group differences for any of the SRH questions. In the elderly and health adults, engagement in physical activity practice has been weakly correlated (*r* = 0.20) with improvements in SRH [58]. Although the benefits of exercise on several aspects of health after stroke have been extensively reported [27], the effects of exercise on SRH have not been investigated.

Considering the need for continuous monitoring of individuals with stroke by the rehabilitation team, general and age-comparative questions should be used along with time-comparative questions for the assessment of SRH after stroke. Furthermore, emotional and physical functions should also be systematically evaluated and targeted during stroke rehabilitation interventions. According to a recently published systematic review on SRH of individuals with stroke, interventions for the improvement of this outcome are still poorly investigated and were not directed to improve emotional and physical functions [5]. Emotional and physical functions are important components of functioning [2] and rehabilitation professionals should apply and integrate approaches of functioning in light of the health condition [4]. Therefore, future investigations on efficacy of interventions directed to improve emotional and physical functions of individuals with chronic stroke, who rate their general health as poor or their comparative health as worse, are mandatory in stroke care.

### Study limitations

Although the estimated sample size was achieved, in general, studies involving SRH are population-based [59]. Only one variable was included to represent emotional/physical functions and personal factors. The variables of the present study were chosen based upon clearly stated criteria, but others should also be investigated. Finally, the results of the present study refer only to individuals with chronic stroke. Therefore, the results cannot be generalized to others at the acute or sub-acute post-stroke stages.

## Conclusion

Despite having chronic disabilities, most of the participants rated their general health as good and their comparative health as better or similar. The three questions used for the assessment of SRH behaved differently. While general and age-comparative questions showed significant differences only for emotional function, time-comparative question showed differences only for physical function. SRH is a recommended health indicator for use within clinical and research contexts, as a simple and comprehensive measure that can be easily obtained by health professionals. However, as already demonstrated with the elderly, the questions should not be used interchangeably. Health professionals should systematically assess SRH of individuals with chronic post-stroke disabilities, using general and age-comparative, along with time-comparative questions, as well as emotional and physical functions.

## Data Availability

The dataset used and/or analyzed during the current study is available from the corresponding author on reasonable request. The data that support the findings of this study are available from Department of Physical Therapy, Universidade Federal de Minas Gerais (Professor Christina Danielli Coelho de Morais Faria), but restrictions apply to the availability of these data, which were used under license for the current study, and so are not publicly available. Data are however available from the authors upon reasonable request and with permission of Municipal Health Secretariat of the cities of Belo Horizonte and Fortaleza.

## Abbreviations

ICF: International Classification of Functioning, Disability, and Health
IQR: Interquartile range
SD: Standard deviation
SF-36: Short-form health survey-36
SPSS: Statistical package for the social sciences
SRH: Self-rated health
WHO: World Health Organization
